# Influence of the COVID-19 Pandemic in Higher Education Courses in the Health Field

**DOI:** 10.17533/udea.iee.v39n2e01

**Published:** 2021-06-15

**Authors:** Vitor Manuel Costa Pereira Rodrigues

**Affiliations:** 1 RN, Ph.D. Research Center in Sports Sciences, Health Sciences and Human Development, CIDESD, Vila Real, Portugal; School of Health, University of Trás-os-Montes e Alto Douro, Vila Real, Portugal. Email: vmcpr@utad.pt Universidade de Trás-os-Montes e Alto Douro University of Trás-os-Montes e Alto Douro Vila Real Portugal vmcpr@utad.pt

It is a fact that no one expected what took place in the world as of the end of 2019. And yes, I am talking about the ill-fated, damned and inconvenient COVID-19, which quickly spread around the globe, having been declared a pandemic by the World Health Organization in March 2020.(1) In the face of such a threat, not virtual, but very real, the need existed to undertake various measures that could interrupt the transmission of the infection by implementing, for example, restrictions on the movement of people, social distancing, use of personal protective equipment, measures of respiratory protocol, social confinement, among others.(2)

Education, namely, higher education, also suffered immensely the consequences of COVID-19, right from the start, because there was a cessation of all classroom teaching activity, practically all over the world, which caused major modifications/alterations in the teaching-learning process.(3) From the outset, closing of Higher Education Institutions, globally, caused severe changes in teaching-learning processes because measures of social distancing and circulation restrictions were imposed.(4,5)

Teaching has, provisionally, become - we hope - remote learning, supported by digital platforms. The need emerged to invest in e-learning platforms and teacher training. However, several important questions arose, such as: were the platforms used for remote learning adequate and safe, and did students have the appropriate technological means at their disposal, in terms of internet access and computer equipment.(6,7) No doubt, students and teachers have had to adapt to the new reality of education: the transition from face-to-face to remote learning. However, the following question can be raised: were teachers trained for remote learning, both in terms of technological skills and in terms of teaching-learning strategies?(8)

Since March 2020, classrooms throughout the globe, engaged in remote learning and some Higher Education Institutions opted for mixed education, that is, theoretical classes in remote learning and practical classes in the classroom. Health students saw their clinical internships/teaching interrupted with clear consequences in the teaching-learning process. “Incoming” and “outcoming” mobility was also severely affected.(9)

The isolation of students, with the consequent lack of social interaction, had effects on stress and anxiety levels,(10) and caused some fear, in the students, of not completing the courses.(11) A major concern arises and may arise, quite naturally, did Higher Education Institutions care about their students, their needs and the teaching-learning process? The answer to this question deserves careful attention and studies at a global level. And to identify the problems/constraints that the COVID-19 pandemic causes/caused in the teaching-learning process to students attending master's courses in health, the best way to know this is to ask the interested parties directly what happened, what problems existed, and what needs were present.

In view of this challenge, and to highlight the relevance of the influence of the COVID-19 pandemic in higher education courses in health, an exploratory and transversal study was conducted with students, in the 2020/2021 academic year, from five courses (Master's in Community Nursing, Master's in Nursing for People in Critical Situation, Master's in Gerontology: Physical Activity and Health in the Elderly, Master's in Health Services Management, and Master's in Biomedical Engineering), seeking to know the opinion of Master's students from courses in the health area on the teaching-learning process during the COVID-19 pandemic.

Data collection took place in February 2021, at a University in northern Portugal, using a self-completed questionnaire created on Google Docs, where data anonymity and confidentiality was ensured and its completion was voluntary. The questionnaire was answered by 58 students, 75.9% female and 24.1% male, with a mean age of 33.6 years and a minimum of 21 years and a maximum of 52 years. Regarding their employment situation, 72.4% of the students were working. A total of 37.9% of the students reported feeling constraints that interfered with the frequency of the course. Among the constraints mentioned, they highlight the accumulation of hours at work, making it impossible to attend all classes, constant changes in work scales and overtime due to the pandemic, physical and mental wear, and difficulty in reconciling work, classes, and family life.

When students were asked if professors provided support documents to conduct the curriculum, 55.2% reported a high/very high satisfaction. As for the feedback professors provide regarding the work used in the evaluation, 53.4% of the students reported medium/low satisfaction. Regarding the adequacy of the required workload and schedule for each curricular unit, 62.1% of students reported medium/low satisfaction. As per student motivation, on the part of professors, for the frequency of curricular units, 53.5% reported medium/low satisfaction. With respect to the adequacy of the curricular units' evaluation processes to the context of the COVID-19 pandemic, 55.2% of the students reported medium/low satisfaction.

We also wanted to know, and taking into account the teaching-learning process, amid the COVID-19 pandemic, aspects that students considered as most negative. Of the most negative aspects mentioned by the students, we highlight the following: the classes and assessments are “online”, lack of practical classes, huge workload to be delivered in very short deadlines, lack of conviviality, interaction, and socialization with colleagues and professors, lack of understanding by professors with students who worked on the COVID-19 front lines and the little flexibility by some professors to adapt syllabus, ways of teaching, and assessment methods.

It is quite evident, in the aforementioned, that the COVID-19 pandemic caused/causes several constraints in students attending master's degrees in health and that it is necessary to minimize/eliminate them. And here the role of Higher Education Institutions and, in particular, teachers is fundamental and indispensable for the adequacy of the teaching-learning process, so that the most vulnerable students are not further unprotected and social and economic inequalities are not intensified.
